# PoDCall: a robust tool for automated droplet classification in DNA‐methylation droplet digital PCR

**DOI:** 10.1002/1878-0261.70105

**Published:** 2025-08-05

**Authors:** Hans Petter Brodal, Heidi Pharo, Hege Marie Vedeld, Guro E. Lind

**Affiliations:** ^1^ Department of Molecular Oncology, Institute for Cancer Research, Norwegian Radium Hospital Oslo University Hospital Norway; ^2^ Department of Biosciences Faculty of Mathematics and Natural Sciences, University of Oslo Norway

We were pleased to see that Neefs *et al*. [1] recently highlighted PoDCall (Positive Droplet Caller) as a tool for automated droplet classification in DNA methylation droplet digital PCR (ddPCR) experiments. As the authors rightly emphasized, automated droplet classification enhances the standardization of DNA methylation analyses and minimizes bias associated with manual scoring. While Neefs *et al*. suggest that PoDCall requires further validation before being widely adopted as a standard tool, we would like to underscore that PoDCall has already undergone extensive testing and evaluation. PoDCall has been successfully used in several studies, including early and accurate detection of cholangiocarcinoma (CCA) among high‐risk patients [2]. In this study, using a DNA methylation‐based biomarker panel, CCA was detected with 100% sensitivity and 90% specificity. Additionally, PoDCall has been utilized in scoring the BladMetrix biomarker test for bladder recurrence, achieving 91% sensitivity and less than 1% false negatives [3]. Notably, the BladMetrix test is currently undergoing validation in a multicenter clinical trial involving around 700 patients, where PoDCall is integral to data analysis.

Furthermore, a recent review, comparing automated methods for droplet calling in dPCR experiments, highlighted PoDCall's robust performance across various datasets [4]. The ability to handle baseline shifts between wells, without relying on control samples, was specifically praised. Importantly, these datasets extended beyond DNA methylation experiments, demonstrating PoDCall's potential for a wider range of ddPCR applications, including copy number alterations.

PoDCall is designed to be user‐friendly and accessible [5]. It is available as an R package with an accompanying Shiny application, requiring no bioinformatic competence. The Shiny app features an intuitive interface and allows visual inspection and manual adjustment of the results. Since its inclusion in Bioconductor (version 3.13; current version 3.20), PoDCall has been actively maintained and regularly updated. The latest edition (version 3.21; April 2025) introduces compatibility with multiplexing up to six targets on the Bio‐Rad QX600 platform.

In summary, PoDCall has been thoroughly tested and validated across various applications, and ongoing development ensures its continued improvement. If implemented as a standard tool in ddPCR experiments, PoDCall could contribute to more efficient data analysis, as well as more consistent and reproducible results.

The communication with the authors of the primary publication is provided as (Data S1).

## Conflict of interest

The authors declare no conflict of interest.

## Author contributions

HPB, HP, HMV, and GEL conceived, wrote, and revised the manuscript. All authors read and approved the final version of the manuscript.

## Supporting information


**Data S1.** Communication with the authors of the primary publication by Neefs *et al*.
