# Supplementary material for: PoDCall: a robust tool for automated droplet classification in DNA‐methylation droplet digital PCR
Source: Mol Oncol. 2025 Aug 5;19(9):2472–3. doi: 10.1002/1878-0261.70105 (PMC12420344; doi:10.1002/1878-0261.70105)
Supplement: Supplementary file 1 — Data S1. Communication with the authors of the primary publication by Neefs et al. [file MOL2-19-2472-s001.docx]

**Communication with the authors of the primary publication by Neefs et al.**

We thank the team of professor Guro Lind for carefully reading our manuscript entitled “Simultaneous detection of eight cancer types using a multiplex droplet digital PCR assay”, published in Molecular Oncology.

The team raised an issue regarding our statement in the discussion part of our manuscript where we highlighted that their tool, PodCall, might need more validation before being adopted as a standard tool. They state in a letter to the editor that their package already underwent extensive testing and validation. However, we respectfully disagree—particularly with regard to the state of validation at the time of our publication—and offer the following arguments:

- At the time of publication, there was only one scientific research paper available that had utilized the PoDCall tool. This aligns with our assessment that the tool had not yet undergone comprehensive validation. Robust validation typically involves multiple, large-scale, multicenter studies. The sole publication, a study on cholangiocarcinoma patients (H.M. Vedeld et al.), featured substantial overlap between the research team and the developers of PoDCall— with the senior author leading both studies. This indicates that PoDCall had not yet been independently or externally validated at that point.
- In their letter to the editor, the authors cite new references in which PoDCall is used as an analysis tool for ddPCR experiments. Specifically, PoDCall was used in a subsequent bladder cancer study (Vedeld et al., *Molecular Oncology*, Nov 2024), and it is currently being used in an ongoing, unpublished multicenter study. Importantly, the results and performance data of PoDCall in both studies were not available to us at the time we wrote our manuscript.
- In their letter to the editor, the authors cite a review (Vynck et al., ClinChem, Sep2023) that highlighted ddPCR analysis methods that do not rely on control samples (such as PodCall) as using (sub-optimal) control samples could lead to baseline shifts and that in certain circumstances, these methods could result in better results. However, in that same paragraph, the authors of the review emphasize that “*such recommendations are purely based on reasoning and have not been validated by comprehensive, numerical method evaluation”*.
- Since the time of our publication (*Neefs et al.*), several updates have been made to the PoDCall package. At the time, version 1.11.1 was available, whereas the current version is 1.16.0. These successive improvements support our view that ongoing testing and validation contributes to the refinement of PoDCall, ultimately enhancing its performance.

We maintain our original position that, at the time of our publication, PoDCall required further validation. It was still a relatively new tool, lacking independent external validation. While we recognize that PoDCall may be useful in specific contexts, we emphasize that careful consideration is essential before incorporating such methods into a standard diagnostic pipeline, where thorough validation is a prerequisite.

Additionally, should this letter to the editor be accepted for publication, we request the opportunity to issue an official response.

We remain available for any further clarification if needed.

Ken Op de Beeck | PhD

Corresponding Author of Neefs et al., “Simultaneous detection of eight cancer types using a multiplex droplet digital PCR assay,” Mol. Oncol., vol. 19, no. 1, pp. 188–203, Jan. 2025, doi: 10.1002/1878-0261.13708.
